# Electrochemical Detection of Uric Acid Based on a Carbon Paste Electrode Modified with Ta_2_O_5_ Recovered from Ore by a Novel Method

**DOI:** 10.1021/acsomega.3c06749

**Published:** 2023-11-30

**Authors:** Shashanka Rajendrachari, Hasan Arslanoglu, Ali Yaras, Shailesh M. Golabhanvi

**Affiliations:** † Department of Metallurgical and Materials Engineering, Faculty of Engineering Architecture and Design, Bartin University, Bartin 74100, Turkey; ‡ Department of Chemical Engineering, Faculty of Engineering, Çanakkale Onsekiz Mart University, Çanakkale 17100, Turkey; § Department of Mechanical Engineering, KLE Dr. M. S. Sheshagiri College of Engineering and Technology, Belagavi 590008, Karnataka, India

## Abstract

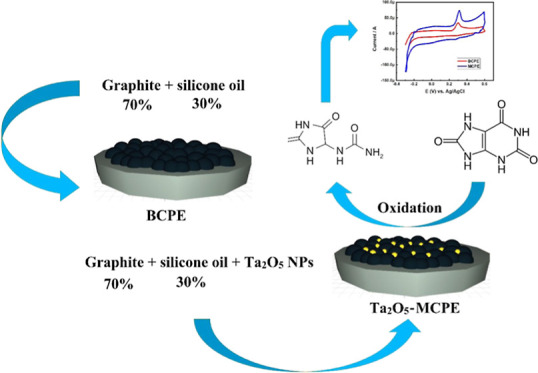

Except for well-known commercial production procedures, this study demonstrates that Ta_2_O_5_ particles can be produced. Through a series of steps, highly pure Ta_2_O_5_ particles (99.45%) were produced from the raw ore. We have electrochemically detected one of the important nitrogenous compounds present in urine, “uric acid”, by a Ta_2_O_5_ particle-modified carbon paste electrode (Ta_2_O_5_-MCPE) using cyclic voltammetry. The prepared electrode has shown excellent current sensitivity at a pH of 6.0 phosphate-buffered solution. We have found that 4 mg Ta_2_O_5_-MCPE has recorded the highest current sensitivity of 75.75 μA. The oxidation peak current was varied with the uric acid concentration in the range from 1 to 5 mM at 4 mg Ta_2_O_5_-MCPE. We have calculated the electrode-active surface area for a bare carbon paste electrode and 4 mg Ta_2_O_5_-MCPE using the Randles–Sevcik equation, and the values were found to be 0.0202 and 0.0450 cm^2^, respectively. On the other hand, the calculated values of limit of detection and limit of quantification were reported as 0.5937 × 10^–8^ M and 1.9791 × 10^–8^ M, respectively, for the prepared 4 mg Ta_2_O_5_-MCPE. The interfere studies revealed that the variation in the electrochemical signal of uric acid in the presence of different metal ions was found to be less than ±5%.

## 1 Introduction

Uric acid (2,6,8-trihydroxy purine) is a very important heterocyclic component of urine and is mainly produced during the metabolic activity of purine.^1^ Regular physiological concentrations of uric acid in blood are 25–80 mg/L and 15–60 mg/L, respectively, for men and women.^2^ Maintaining the accurate level of uric acid in the body is quite difficult due to factors like food and genetic variations.^3^ Seafood and meat can increase the amount of uric acid in blood.^4^ The abnormal amount of uric acid in humans can cause severe diseases like gout, hypertension, hyperuricemia, diabetes, heart failure, and Lesch–Nyhan syndrome.^5,6^ Therefore, frequent monitoring of uric acid in the body with a sensitive and selective method is very important. Techniques like liquid chromatography,^7^ titration,^8^ capillary electrophoresis,^9^ and spectrophotometric^10^ are employed in the determination of uric acid. However, the high use of chemicals is just one of the many drawbacks of these methods. Due to their comparatively low equipment costs, quick response times, simple operations, time savings, and real-time detection under in situ conditions, electrochemical methods have become popular among researchers.^11–14^

There are many superior properties of carbon paste electrodes (CPEs), which are usually preferred for electrochemical studies: ease of modification with aqueous/anhydrous matrices, high conductivity and adsorption capacity, low residual current, new repeatability, and wide potential window.^15,16^ In recent years, studies on use of CPEs modified with various metal oxides for electrochemical applications have been reported.^17–20^ Undoubtedly, the goal of these modifications is to maximize selectivity in order to enhance the electrode performance. In this context, one of these metal oxide types used is tantalum oxide (Ta_2_O_5_). High chemical and thermal stability and high pH sensitivity of Ta_2_O_5_, a transition metal oxide compound, are among its most significant characteristics.^21,22^ Additionally, Ta_2_O_5_-based carbon and nitrogen molecules can function as catalysts for reduction processes in polymer electrolyte fuel cells.^23^ In addition, it is also known that metal-doped Ta_2_O_5_ films have good electrochemical reversibility and catalytic performances.^24^ Ta_2_O_5_ is a crucial substance for the design and development of electrochemical sensors because of all of these characteristics. On the other hand, literature data show that CPE modification with Ta_2_O_5_ and its electrochemical application are quite limited. One of them is that the increased surface area of CPE modified with Ta_2_O_5_ boosts its electrochemical catalytic activity. A fabricated sensor enabled the determination of flavonoids with repeatable and dependable findings.^25^ In another, chrysin and baicalein determinations were carried out using electrodes that had been modified with Ta_2_O_5_ particles and chitosan. In both specimens, the linear range was found to be 0.08–4.0 μM. The produced sensor demonstrated good sensitivity and exceptional stability, with detection limits for chrysin and baicalein measured at 0.03 and 0.05 μM, respectively.^12^

When the aforementioned studies are taken into consideration, it becomes evident that Ta_2_O_5_ powders employed are analytically pure commercial items. Difference and innovative aspect of the present study is that Ta_2_O_5_ powders are obtained from natural ore as a result of some processes and evaluated in the determination of uric acid levels. This scope of this paper, frequent monitoring of uric acid in the body, is very important, and therefore, we have developed a simple, economic, stable, and highly sensitive Ta_2_O_5_-MCPE to determine uric acid. Fabricated electrode has shown excellent selectivity and antifouling characteristics in determining uric acid.

## 2 Materials and Methods

### 2.1 Raw Material and Characterization

Ore containing Ta_2_O_5_ was brought from a mine in the Republic of Congo. The chemical composition of raw ore is given in Table 1. According to the data, the ore structure is highly complex. Ta_2_O_5_ is not only the substance present; Nb_2_O_5_, Fe_2_O_3_, SnO_2_, and Al_2_O_3_ are also present at significant levels. Therefore, it is obvious that a stepwise procedure is required to produce Ta_2_O_5_ of high purity.

**Table 1:** Chemical Composition of Raw Ore Used in Experiments

components	%
Ta_2_O_5_	17.25
Nb_2_O_5_	15.76
Fe_2_O_3_	10.00
SnO_2_	10.61
Al_2_O_3_	9.12
MnO	7.72
SiO_2_	9.52
TiO_2_	8.21
others	1.21
loss ignition	10.6

### 2.2 Methods

**Figure 1:**
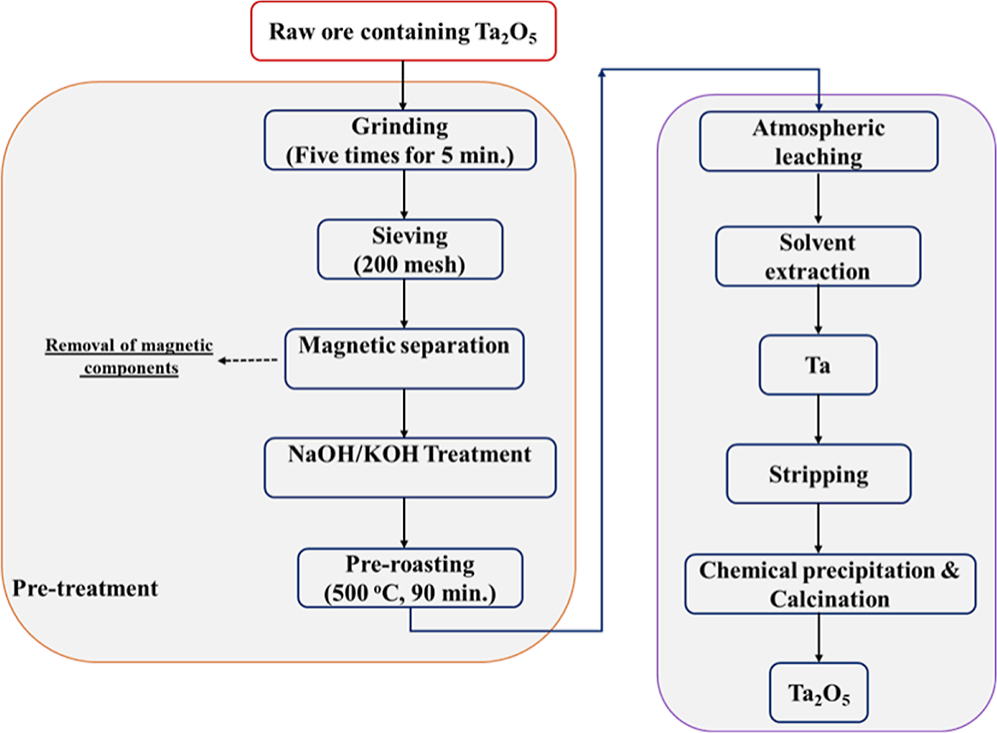
Flowchart of the Ta_2_O_5_ recovery process from raw ore.

It is vital to enhance the sample surface area and remove impurities in order to increase leaching efficiency. Consequently, following grinding and sieving, magnetic separation was used to separate magnetic components. Then, the sample was mixed with a NaOH/KOH (99 and 100% purity) mixture, 1.5 times its weight, for 10 min before roasting. Roasting was carried out at 500 °C for 90 min using a chamber oven. The roasted specimen was placed inside the reactor and put through following leaching procedures: liquid/solid ratio 6–8 mL/g, leaching temperature 80–90 °C, leaching time 60–90 min, stirring speed 600 rpm, and pH in the range of 9 and 10. The extraction efficiency of Ta was found to be 94% under these leaching conditions. Following that, in the solvent extraction procedure (liquid/liquid ratio 1.25 mL/mL, temperature 40–50 °C, leaching time 20–30 min, and stirring speed 200 rpm), pure methyl isobutyl ketone and kerosene were employed. Using an incubator, Ta was stripped under the following circumstances: liquid/liquid ratio of 1.0 mL/mL, temperature of 20 °C, stripping time of 30 min, and stirring speed of 200 rpm. Finally, after chemical precipitation at pH 9, calcination was carried out at 700 °C for 90 min. After all these procedures, the purity of Ta_2_O_5_ is 99.45%. The flow diagram of the proposed process for the recovery of Ta_2_O_5_ from raw ore is presented in Figure 1.

**Figure 2:**
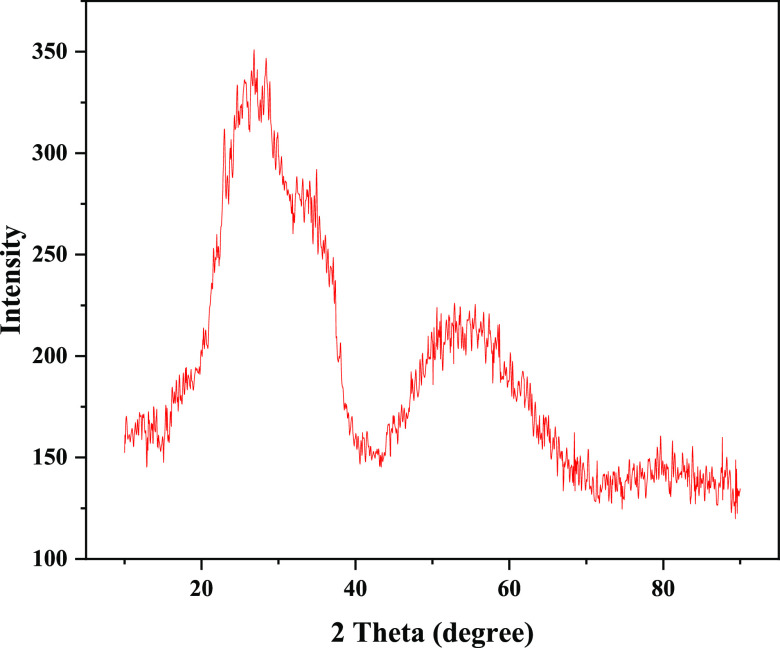
XRD pattern of the final product.

**Figure 3:**
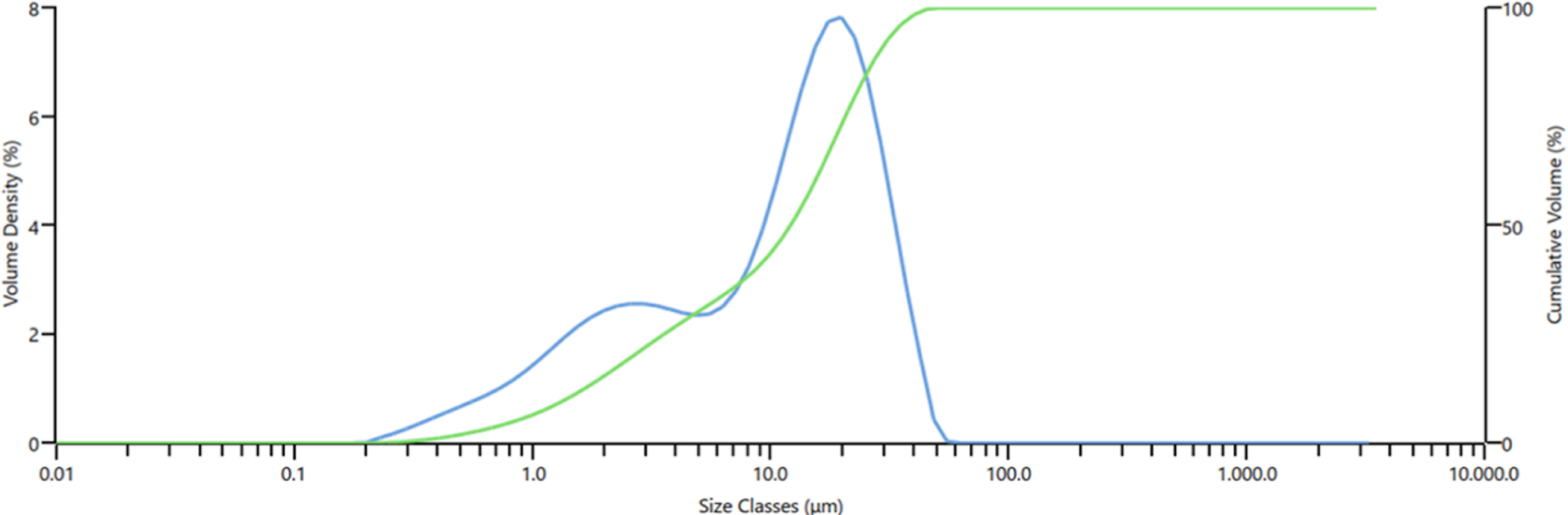
Particle size distribution of Ta_2_O_5_ from ore.

**Figure 4:**
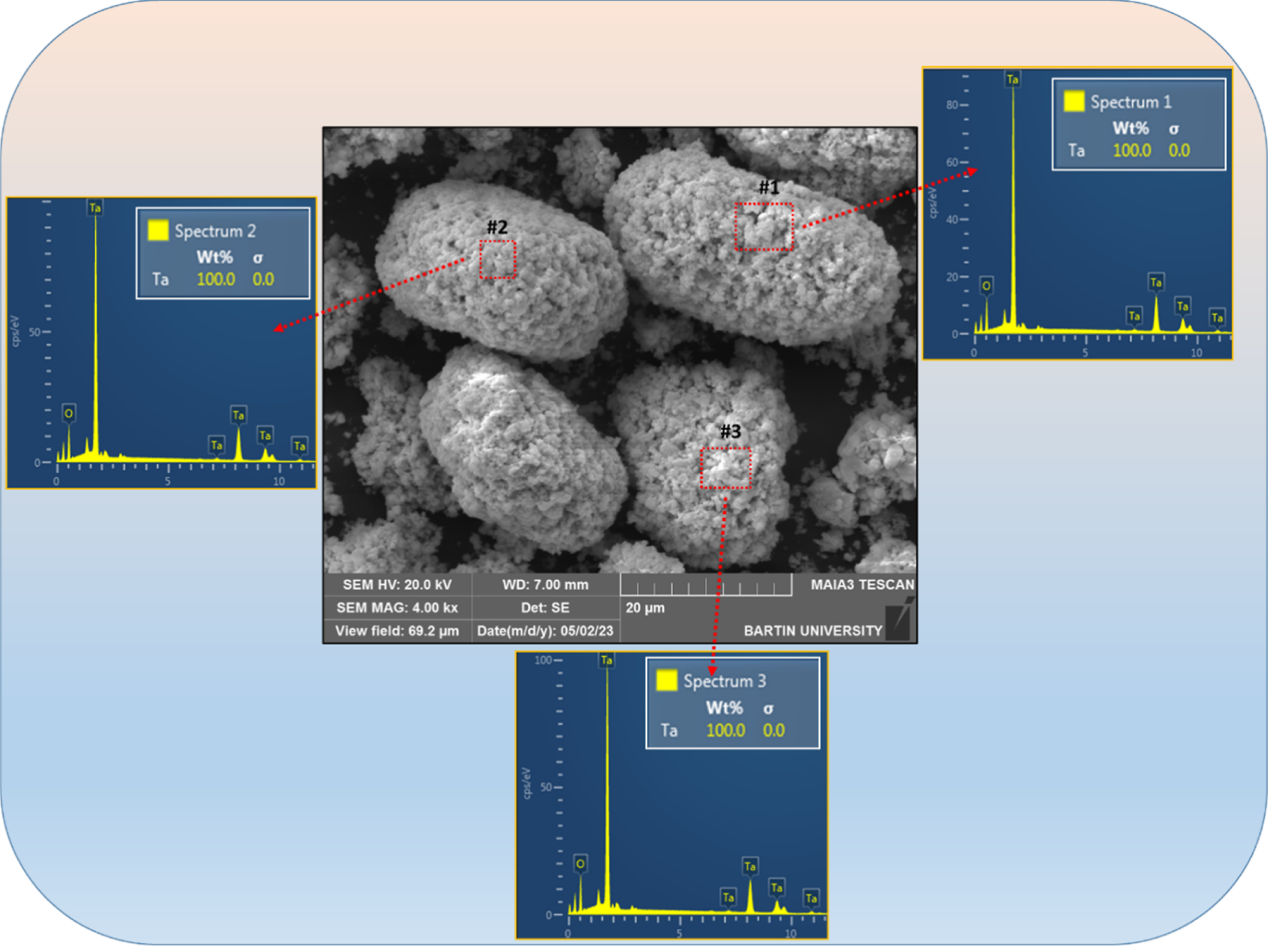
SEM–EDX analysis of Ta_2_O_5_ of raw ore.

A significant crystalline peak is not present in the structure of the final product according to X-ray diffraction (XRD) data in Figure 2. In other words, it is understood that the product has an amorphous structure and no particular crystallization. XRD data are consistent with previous studies.^26,27^ The chemical composition of the finished product was examined using inductively coupled plasma optical emission spectrometry (ICP-OES) as a result of multistep processes (Table 2). Despite having a complicated structure, the ore, according to analysis data, yields a 99.45% (Ta_2_O_5_) pure final product. As can be seen from Figure 3, 10, 50, and 90% of the produced Ta_2_O_5_ particles have particle sizes of 1.38 μm, 12.1, and 28.9 μm, respectively. Scanning electron microscopy (SEM–EDX) results indicate that the produced Ta_2_O_5_ particles are agglomerated, and they contain Ta element (Figure 4).

**Table 2:** ICP-OES Analysis Results of the Final Product

components	%
Ta_2_O_5_	99.45
Nb_2_O_5_	11.8 mg/L
Fe_2_O_3_	6.3 mg/L
SnO_2_	4.6 mg/L
Al_2_O_3_	6.1 mg/L
PbO	1.4 mg/L
MgO	3.2 mg/L
CaO	5.6 mg/L
MnO	4.9 mg/L
SiO_2_	3.8 mg/L
TiO_2_	2.7 mg/L
ZnO	0.12 mg/L
As_2_O_3_	0.28 mg/L
WO_3_	0.11 mg/L

### 2.3 Fabrication of Carbon Electrodes

A bare carbon paste electrode (BCPE) was prepared by hand-grinding the 70% graphite powders and 30% silicone oil in an agate mortar for 30 min to obtain a homogeneous mixture of carbon paste. Similarly, we have also hand-ground the same composition of graphite powders and silicone oil along with different concentrations (2, 4, 6, 8, and 10 mg) of the Ta_2_O_5_ nanoparticles individually and separately for 30 min in an agate mortar. For electrochemical studies, we have used Zive SP1 galvanostat/potentiostat, which is a three-electrode system composed of a working electrode (prepared carbon paste electrodes), a platinum counter electrode, and a reference electrode, respectively. The prepared carbon paste was inserted into a 3 mm cavity present in a polymer working electrode connected with a copper wire at the end to study the current response. We have used all the fabricated, different concentrated Ta_2_O_5_-modified carbon paste electrodes and recorded their current response in determining 1 mM uric acid.

## 3 Results and Discussion

### 3.1 Electrochemical Determination of Uric Acid

#### 3.1.1 Optimizing Electrochemical Detection at Different Ta_2_O_5_-MCPE

**Figure 5:**
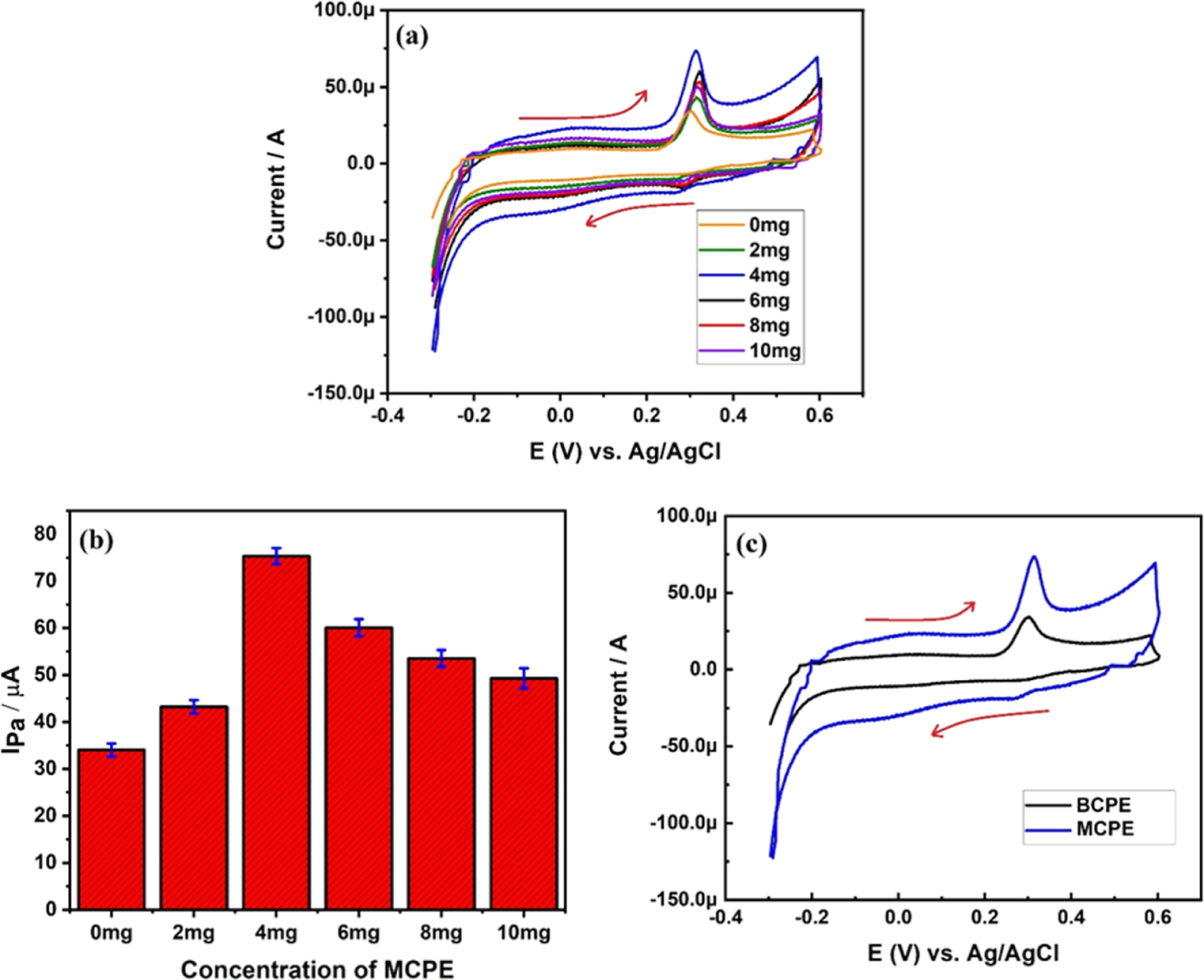
(a) Cyclic voltammogram of 1 mM uric acid at a pH of 6 PBS at different concentrations of the modifier; (b) plot of various concentrations of Ta_2_O_5_ nanoparticles in MCPE and their respective anodic peak current; and (c) cyclic voltammogram of 1 mM uric acid at BCPE and 4 mg Ta_2_O_5_-MCPE.

Excellent current sensitivity mainly depends on choosing the correct concentration of the modifier. Therefore, we have recorded current response during electro-oxidation of 1 mM uric acid at a pH of 6 phosphate-buffered solution (PBS) using cyclic voltammetry at different concentrations of the modifier as shown in Figure 5a. We have also plotted anodic peak currents obtained, respectively, for BCPEs 2, 4, 6, 8, and 10 mg Ta_2_O_5_-MCPE as shown in Figure 5b.

Figure 5b depicts that 4 mg Ta_2_O_5_-MCPE has shown a maximum anodic peak current of 75.75 μA compared to BCPE, which has depicted only 34.05 μA of peak current. This proves the sensitivity of fabricated Ta_2_O_5_-MCPE, which can determine uric acid even in low concentrations with excellent sensitivity. The surface area of BCPE and MCPE is comparatively different, and this is the main reason for increased sensitivity of MCPE. To study this in detail, we have calculated the electrode surface area by the Randles–Sevcik equation^28,29^ as follows
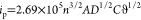
1

The calculated active surface area for BCPE and 4 mg Ta_2_O_5_-MCPE is found to be 0.0202 and 0.0450 cm^2^, respectively. The increased surface area of MCPE increases the reactive sites along with surface roughness, as a result of which the interaction of electrons between the electrode and analyte increases significantly. Therefore, based on the results, we have used 4 mg Ta_2_O_5_-MCPE for further studies. Figure 5c depicts the cyclic voltammetric curve of 1 mM uric acid for BCPE and 4 mg Ta_2_O_5_-MCPE, respectively. From the figure, we can clearly see the differences in anodic peak current during electro-oxidation of uric acid in PBS of pH 6.

#### 3.1.2 Effect of pH

**Figure 6:**
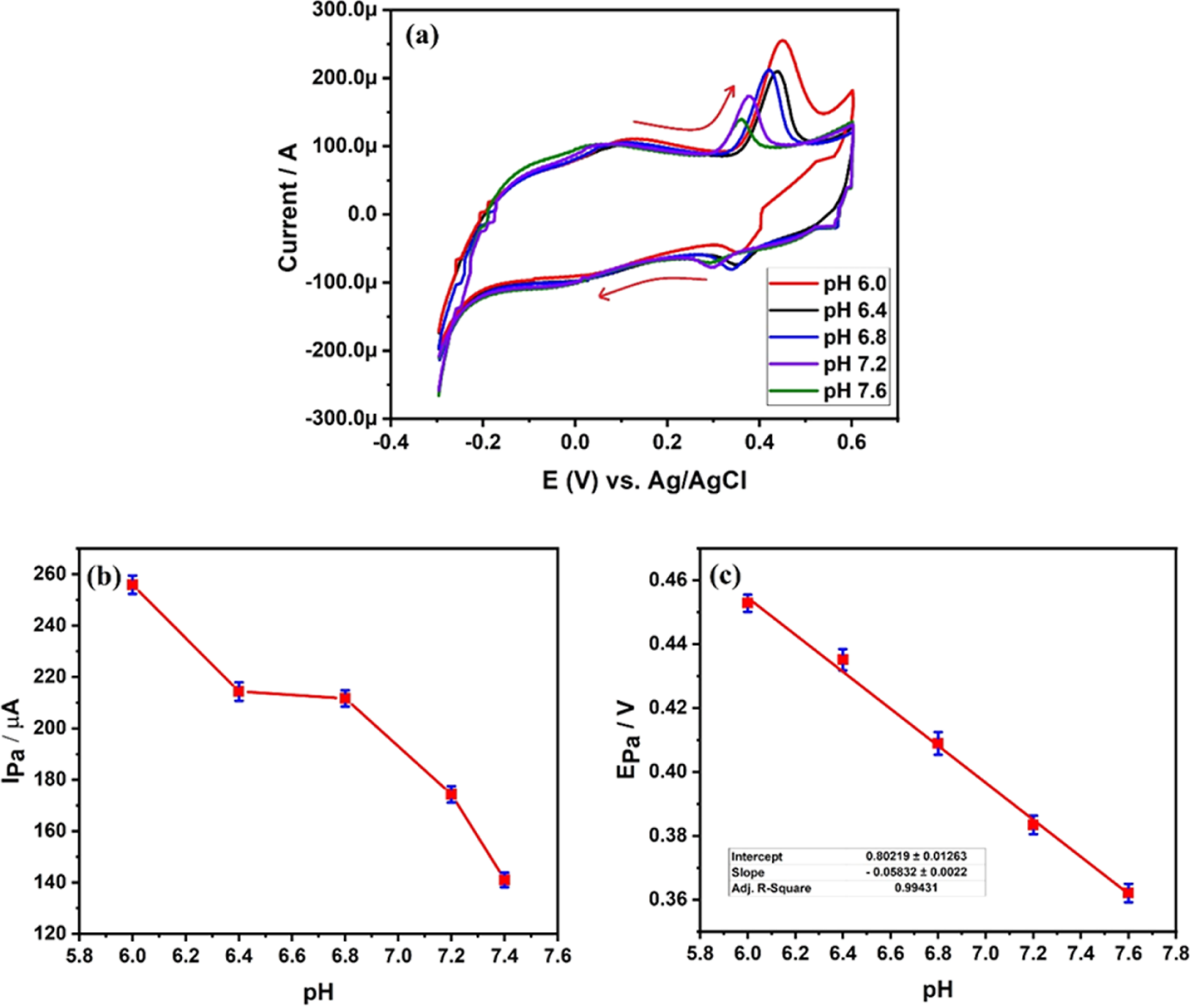
(a) Cyclic voltammogram of 4 mg Ta_2_O_5_-MCPE in 1 mM uric acid solution at different pHs with a scan rate of 0.1 V s^–1^, graph of pH vs (b) *I*_pa_ and (c) *E*_pa_ at 1 mM uric acid.

**Figure 7:**
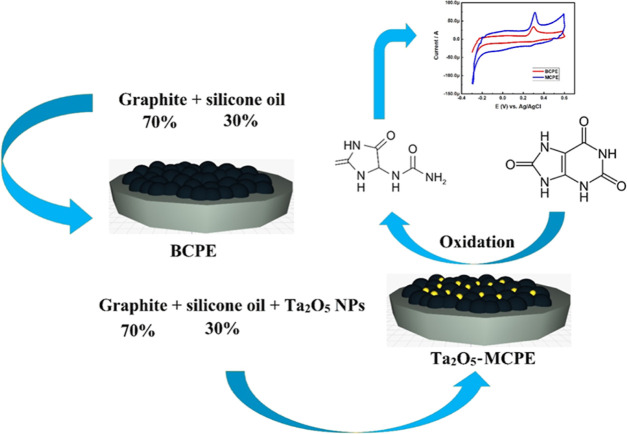
Electrochemical processes of Ta_2_O_5_-MCPE for detection of uric acid.

The investigation of the electrochemical response of the electrode in pH is very crucial to understand the number of electrons and protons involved in the electrode reaction. This also has an effect on the stability of the electrode because few analytes are reactive in acidic pH and few of them in basic pH. Therefore, we have studied the current sensitivity of fabricated 4 mg Ta_2_O_5_-MCPE against different pHs from 6 to 7.6, and their cyclic voltammetry curves are shown in Figure 6a. The anodic peak current of uric acid decreased with an increase in pH from 6 to 7.6, as shown in Figure 6b. Maximum current sensitivity for uric acid was recorded at a pH of 6, and therefore, we have recorded all voltammetric measurements at this pH. We have also plotted a graph of anodic peak potential at different pHs as shown in Figure 6c. From this plot, it is noted that the anodic peak potential is shifting toward a more negative potential with an increase in pH value from 6 to 7.6. This indicates the participation of protons in the electrode reaction. The graph of variation of the anodic peak potential with pH is linear and follows the equation *E*_p_ (*V*) = 0.8021–(0.05832) pH (V/pH) (*R*^2^ = 0.9943), with an excellent linear regression coefficient (*R*^2^). This confirms the dependency of electron transfer during oxidation and reduction of uric acid on protonation.^30–34^ Further, we have also calculated the number of protons (*m*) involved in oxidation and reduction reaction based on a graph of pH versus *E*_p_ by the below equation
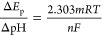
2where *R* is the gas constant, *T* is the temperature in kelvin, *n* is the number of electrons, and *F* is the Faraday constant. The number of protons (*m*) involved in electrode reaction is found to be 1.966, and the value is nearly equal to 2. Therefore, the electrochemical redox reaction of uric acid is taking place with involvement of two electrons and two protons. Figure 7 illustrates the electrochemical oxidation reaction process of uric acid on the surface of Ta_2_O_5_-MCPE.

#### 3.1.3 Effect of Scan Rate

**Figure 8:**
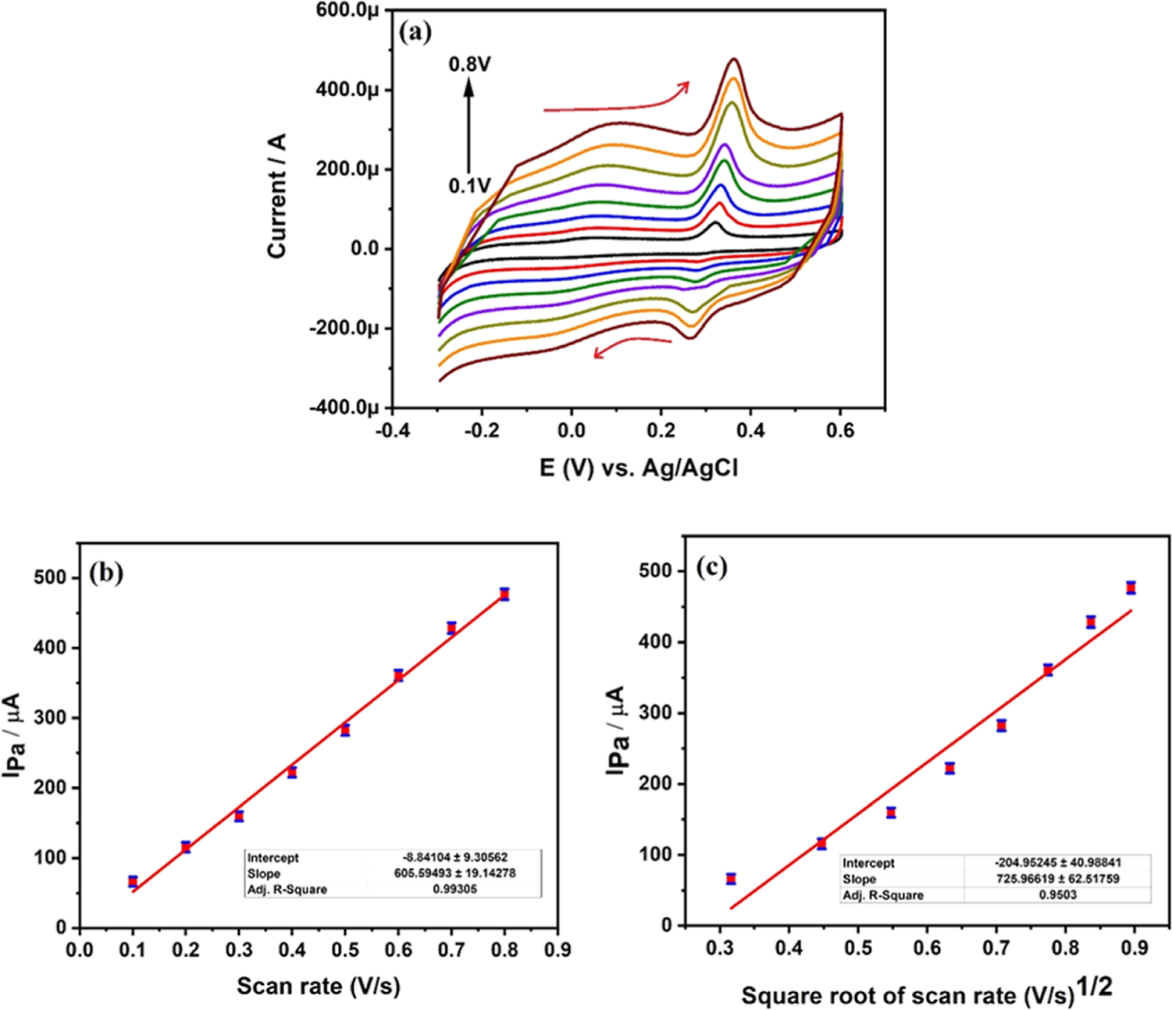
(a) Cyclic voltammetry response of uric acid at different scan rates from 0.1 to 0.8 V; (b) plot of *I*_pa_ vs scan rate, and (c) *I*_pa_ vs square root of scan rate at a pH of 6 PBS.

Investigating the current response at different scan rates is very important to understand the type of electrochemical reaction and also the stability of the electrode.^35–38^ This study reveals whether the electrochemical reaction is adsorption controlled or diffusion controlled. Hence, we reported cyclic voltammetry response of uric acid at different scan rates from 0.1 to 0.8 V as shown in Figure 8a. From the figure, it is confirmed that an increase in scan rate increases electro-oxidation of uric acid in a PBS of pH 6 along with a positive shift of potential. To understand the dependence of fabricated MCPE and uric acid molecules in detail, we have plotted a graph of scan rate versus anodic current and also square root of scan rate versus anodic peak current, respectively, depicted in Figure 8b,c. There is a linear increase in anodic peak current in both graphs, indicating the fast and direct electron transfer between uric acid and surface of 4 mg Ta_2_O_5_-MCPE. This confirms strong binding of uric acid molecules on MCPE. We have calculated correlation coefficient for graphs Figure 8b,c to understand the type of electrode reaction involved, and their values were found to be 0.9932 and 0.9528, respectively. This confirms that the type of electrode reaction is diffusion controlled.

#### 3.1.4 Effect of Concentration of Uric Acid

**Figure 9:**
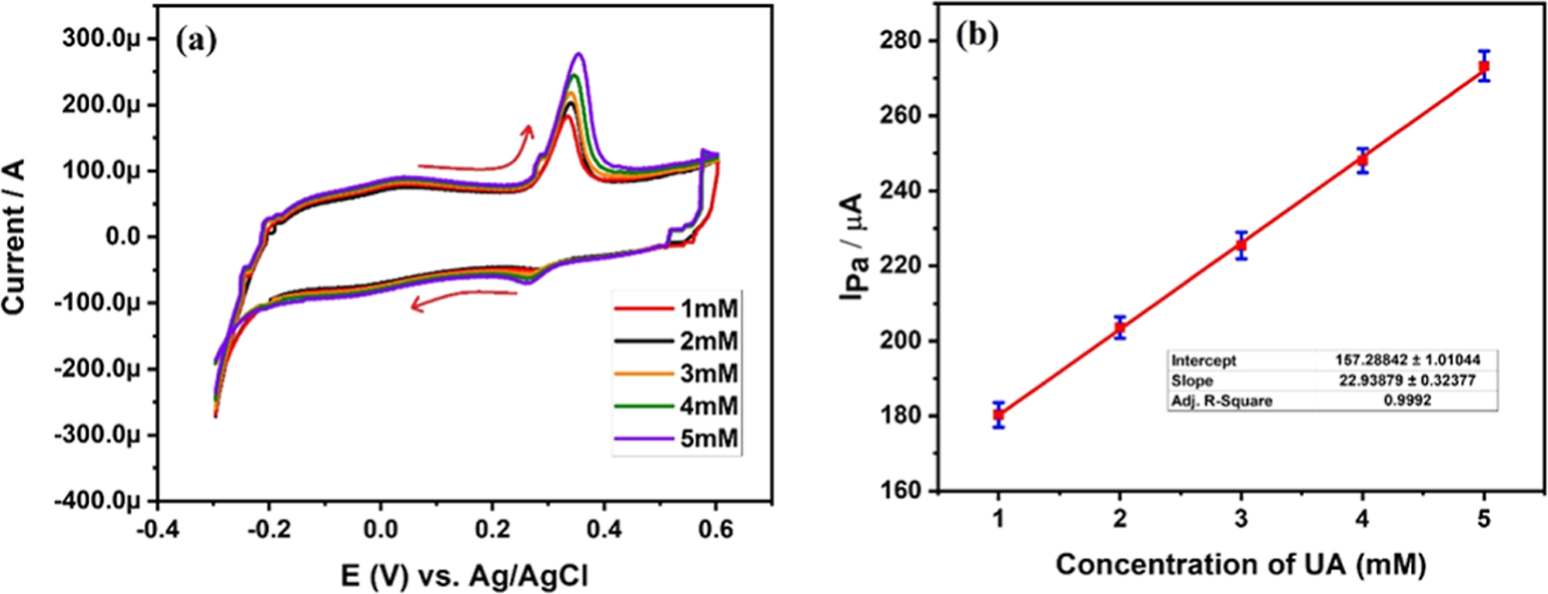
(a) Cyclic voltammogram curve at different concentrations of uric acid. (b) Plot of *I*_pa_ versus concentration of uric acid.

In general, the current response of the analyte depends mainly on its concentration.^39–42^ If the concentration of the analyte is greater, we can expect maximum current sensitivity and vice versa with low concentration. To understand the stability and effectiveness of fabricated MCPE, we have studied the effect of uric acid concentration on electro-oxidation of uric acid at a PBS of pH 6. Figure 9a depicts a cyclic voltammogram of uric acid at different concentrations starting from 1 to 5 mM, respectively. From cyclic voltammogram, it is confirmed that the anodic peak current response is increasing from 180.87 to 273.82 μA. Figure 9b depicts a graph of concentration of uric acid versus anodic peak current, and it confirms a linear increase in anodic peak current with increasing concentration of uric acid. The increased current sensitivity is due to increased molecules of uric acid in the PBS electrolyte, which in turn increases the interaction of uric acid molecules and electron movement between the electrode surface and electrolyte. Therefore, we recorded the maximum anodic peak current at a higher concentration of 5 mM uric acid with a correlation coefficient of 0.9986.

Limit of detection (LOD) and limit of quantification (LOQ) of 4 mg Ta_2_O_5_-MCPE were determined using the slope of Figure 9b and the standard deviation of blank voltammogram (give cycles and the standard deviation obtained was 2.221 × 10–8), respectively, using eqs 3 and (4) as follows^43^
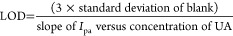
3
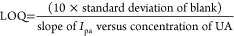
4

The calculated values of LOD and LOQ were found to be 289.69 × 10–^8^ M and 965.65 × 10–^8^ M, respectively, for the prepared 4 mg Ta_2_O_5_-MCPE. Table 3 depicts a comparison of the LOD of Ta_2_O_5_-MCPE for UA with other reported electrodes.

**Table 3:** Comparison of Ta_2_O_5_-MCPE Performance with Other Electrodes Reported for UA

modifier used	analyte detected	LOD (M)	interference	references
sodium dodecyl sulfate	uric acid	3.3 × 10^–^^7^	dopamine and ascorbic acid	(44)
poly(threonine)	uric acid	2.9 × 10^–^^7^	tyrosine and ascorbic acid	(45)
poly(proline)	uric acid	4.7 × 10^–^^6^	dopamine	(46)
octoxynol-9 (OXL-9)	uric acid	1.4 × 10^–^^6^	estriol and dopamine	(47)
poly(glutamic acid)	uric acid	4.3 × 10^–^^7^	norepinephrine and ascorbic acid	(48)
multiwalled carbon nanotube	uric acid	2.9 × 10^–^^8^	epinephrine and ascorbic acid	(49)
poly(calmagite)	uric acid	1.0 × 10^–^^8^	ascorbic acid	(50)
TX-100	uric acid	5.0 × 10^–^^6^	norepinephrine and ascorbic acid	(51)
2-hydroxybenzimidazole	uric acid	5.1 × 10^–^^6^	adrenaline	(52)
Ta_2_O_5_ particles	uric acid	0.5937 × 10^–^^8^	Fe^2+^, Fe^3+^, Na^+^, Mg^2+^, K^+^, and Cu^2+^	present paper

#### 3.1.5 Effect of Interferents

**Figure 10:**
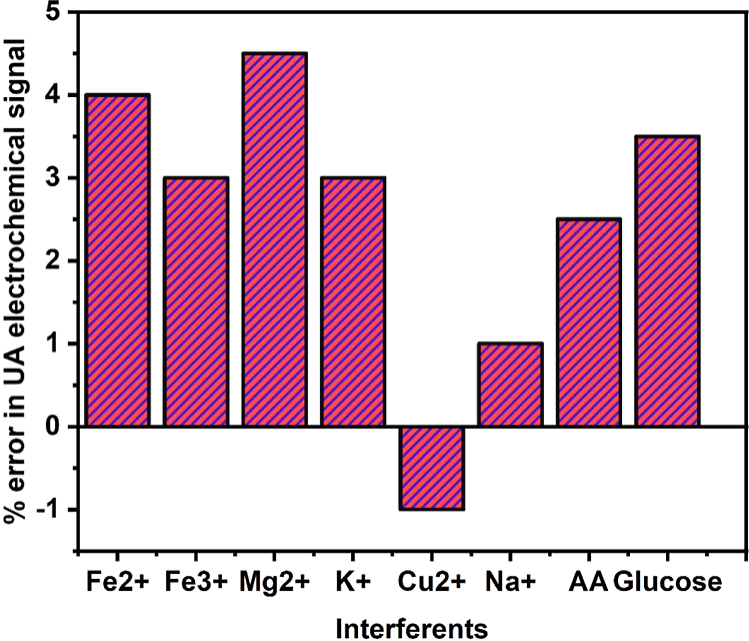
Plot of interferents versus % of error in electrochemical signal of uric acid.

Electrochemical determination of 1 mM uric acid at 4 mg Ta_2_O_5_-MCPE was carried out in the presence of few interfering metal ions like Fe^2+^, Fe^3+^, Na^+^, Mg^2+^, K^+^, Cu^2+^, glucose, and ascorbic acid (AA) to investigate the influence of these metal ions in determining the uric acid analyte and also to check whether any substantial difference in the current response or shifting of potential. From the experiment, it was confirmed that there is no significant variation in the electrochemical signal of 1 mM uric acid even in the presence of interfere ions. This confirms the stability and selectivity of fabricated electrode Ta_2_O_5_-MCPE to determine uric acid. The variation in electrochemical signal recorded is less than ±5% as shown in Figure 10.

#### 3.1.6 Repeatability, Stability, and Reproducibility of 4 mg Ta_2_O_5_-MCPE

The stability, reproducibility, and repeatability of 4 mg Ta_2_O_5_-MCPE were determined by electro-oxidizing UA in a phosphate-buffered solution of pH of 6 and a scan rate of 0.1 V/s. Both repeatability and reproducibility tests were carried out at least five times each by changing the electrode and electrolyte, respectively. The determined relative standard deviation values for reproducibility and repeatability were found to be 2.95 and 2.1%, respectively. Therefore, the fabricated Ta_2_O_5_-MCPE can be repeated and reproduced without much deviation in the original value of the oxidation peak current during the detection of UA. We have also investigated the stability of the prepared Ta_2_O_5_-MCPE by running 50 cycles for detection of UA in a pH 6 solution. We have recorded the initial and final oxidation peak current during the electro-oxidation of UA to determine the degree of stability of the fabricate electrode. We have obtained stability value 95.47%, and this value confirms that the fabricated Ta_2_O_5_-MCPE has shown outstanding stability during electrochemical determination of UA.

## 4 Conclusions

Ta_2_O_5_ production procedures and uses are now crucial because of the growing popularity of Ta_2_O_5_. In the context of this paper, high-purity Ta_2_O_5_ powders were made from natural ore using a variety of techniques, including atmospheric leaching, solvent extraction, stripping, chemical precipitation, and calcination. Modified carbon electrodes made from 99.45% pure Ta_2_O_5_ particles demonstrated sensitive and selective uric acid measurement capabilities.

The reported electrode has depicted excellent current sensitivity at a 4 mg modifier concentration. The calculated electrode-active surface area reveals that the Ta_2_O_5_-MCPE surface area is at least 2 times the surface area of BCPE. This shows the importance of modification to determine the analyte very effectively and efficiently. LOD and LOQ were calculated to be 0.5937 × 10^–8^ M and 1.9791 × 10^–8^ M, respectively, for the prepared 4 mg Ta_2_O_5_-MCPE. We have also reported that the variation in the electrochemical signal of uric acid in the presence of K, Fe, Cu, and Mg metal ions was found to be less than ±5%. This confirms the selectivity of the electrode in which the interfere metal ions do not influence the electrochemical signal of uric acid.
